# Antimicrobial Resistance and Genetic Lineages of *Staphylococcus aureus* from Wild Rodents: First Report of *mec*C-Positive Methicillin-Resistant *S. aureus* (MRSA) in Portugal

**DOI:** 10.3390/ani11061537

**Published:** 2021-05-25

**Authors:** Vanessa Silva, Sofia I. Gabriel, Sofia B. Borrego, Maria Teresa Tejedor-Junco, Vera Manageiro, Eugénia Ferreira, Lígia Reis, Manuela Caniça, José L. Capelo, Gilberto Igrejas, Patrícia Poeta

**Affiliations:** 1 Microbiology and Antibiotic Resistance Team (MicroART), Department of Veterinary Sciences, University of Trás-os-Montes and Alto Douro (UTAD), 5000-801 Vila Real, Portugal; vanessasilva@utad.pt; 2 Department of Genetics and Biotechnology, Functional Genomics and Proteomics’ Unit, University of Trás-os-Montes and Alto Douro, 5000-801 Vila Real, Portugal; gigrejas@utad.pt; 3 Functional Genomics and Proteomics Unit, University of Trás-os-Montes and Alto Douro (UTAD), 5000-801 Vila Real, Portugal; 4 Associated Laboratory for Green Chemistry (LAQV-REQUIMTE), University NOVA of Lisbon, 2829-516 Lisbon, Portugal; 5 Veterinary and Animal Research Centre, Associate Laboratory for Animal and Veterinary Science (AL4AnimalS), University of Trás-os-Montes and Alto Douro (UTAD), 5000-801 Vila Real, Portugal; 6 CESAM—Centro de Estudos do Ambiente e do Mar, Departamento de Biologia da Universidade de Aveiro, Campus Universitário de Santiago, 3810-193 Aveiro, Portugal; sofiagabriel@ua.pt; 7 Departamento de Biologia Animal, Faculdade de Ciências, Universidade de Lisboa, Campo Grande, 1749-016 Lisboa, Portugal; 8 Direção Regional da Agricultura, Secretaria Regional da Agricultura e Desenvolvimento Rural, Quinta de São Gonçalo, 9500-343 Ponta Delgada, Portugal; Sofia.B.Borrego@azores.gov.pt; 9 Research Institute of Biomedical and Health Sciences, University of Las Palmas de Gran Canaria, 35001 Canary Islands, Spain; mariateresa.tejedor@ulpgc.es; 10 Department of Clinical Sciences, University of Las Palmas de Gran Canaria, 35001 Canary Islands, Spain; 11 National Reference Laboratory of Antibiotic Resistances and Healthcare Associated Infections (NRL-AMR/HAI), Department of Infectious Diseases, National Institute of Health Dr Ricardo Jorge, 1649-016 Lisbon, Portugal; vera.manageiro@insa.min-saude.pt (V.M.); Eugenia.Ferreira@insa.min-saude.pt (E.F.); ligia.reis@insa.min-saude.pt (L.R.); manuela.canica@insa.min-saude.pt (M.C.); 12 Centre for the Studies of Animal Science, Institute of Agrarian and Agri-Food Sciences and Technologies, Oporto University, 4051-401 Oporto, Portugal; 13 BIOSCOPE Group, LAQV@REQUIMTE, Chemistry Department, Faculty of Science and Technology, NOVA University of Lisbon, 2825-466 Almada, Portugal; jlcm@fct.unl.pt; 14 Proteomass Scientific Society, 2825-466 Costa de Caparica, Portugal

**Keywords:** *mec*C, MRSA, wild rodents, *S. aureus*

## Abstract

**Simple Summary:**

*Staphylococcus aureus* is present in the microbiota of both humans and some animal species, being recognized as one of the most important opportunistic human pathogens. *S. aureus* is responsible for causing a variety of infections. Methicillin-resistant *S. aureus* (MRSA) is particularly important, as it is becoming increasingly prevalent in the population. MRSA has been increasingly reported among wild free-living animals which may impose a public health concern due to its zoonotic potential. To investigate the prevalence and antimicrobial resistance of *S. aureus* and MRSA in wild synanthropic rodent populations, we conducted this study on 204 rodents captured in port areas in Portugal. The antimicrobial resistance was investigated in all isolates as well as virulence genes and genetic lineages. Thirty-eight *S. aureus* were isolated. The results showed that six MRSA were detected with particularly interesting *mec*C-carrying MRSA isolates which had not yet been found in Portugal. A low frequency of antibiotic resistance and virulence genes was observed among the isolates. Nevertheless, a high diversity of clonal lineages was detected among *S. aureus* some of which are associated with livestock.

**Abstract:**

The frequent carriage of *Staphylococcus aureus*, including methicillin-resistant *S. aureus* (MRSA), by wild animals along with its zoonotic potential poses a public health problem. Furthermore, the repeated detection of the *mec*A gene homologue, *mec*C, in wildlife raises the question whether these animals may be a reservoir for *mec*C-MRSA. Thus, we aimed to isolate *S. aureus* and MRSA from wild rodents living in port areas and to characterize their antimicrobial resistance and genetic lineages. Mouth and rectal swab samples were recovered from 204 wild rodents. The samples were incubated in BHI broth with 6.5% of NaCl and after 24 h at 37 °C the inoculum was seeded onto Baird-Parker agar, Mannitol Salt agar and ORSAB (supplemented with 2 mg/L of oxacillin) plates. Species identification was confirmed by MALDI-TOF MS. The antimicrobial susceptibility testing was performed by the Kirby–Bauer disc diffusion method against 14 antibiotics. The presence of virulence and resistance genes was performed by PCR. The immune evasion cluster (IEC) system was investigated in all *S. aureus*. All isolates were characterized by MLST, *spa*- and *agr* typing. From 204 samples, 38 *S. aureus* were isolated of which six MRSA were detected. Among the six MRSA isolates, three harbored the *mec*C gene and the other three, the *mec*A gene. All *mec*C-MRSA isolates were ascribed to sequence type (ST) 1945 (which belongs to CC130) and *spa*-type t1535 whereas the *mec*A isolates belonged to ST22 and ST36 and *spa*-types t747 and t018. Twenty-five *S. aureus* were susceptible to all antibiotics tested. *S. aureus* isolates were ascribed to 11 MLST and 12 *spa*-types. *S. aureus* presents a great diversity of genetic lineages in wild rodents. This is the first report of *mec*C-MRSA in Portugal.

## 1. Introduction

*Staphylococcus aureus* is a major opportunistic pathogen that can colonize and infect humans and animals. *S. aureus* is found as part of the skin and mucous membranes of humans and some animal species. This pathogen is responsible for various types of infections, such as skin and soft tissue infections and toxin-mediated syndromes as well as life-threatening infections such as bacteremia, osteomyelitis and endocarditis [1]. *S. aureus* has the ability to easily acquire antimicrobial resistance determinants and has an extensive number of virulence factors which are used to establish and maintain infection [2]. *S. aureus* has been isolated from several animals, including pets and livestock, which are in close contact with humans, and wild animals [3]. Methicillin-resistant *Staphylococcus aureus* (MRSA) is a major clinical problem in hospitals worldwide [4]. MRSA was initially restricted to the hospital environment causing several types of nosocomial infections and it was named hospital-acquired MRSA (HA-MRSA). Later, MRSA was found in individuals in human communities (community-associated (CA)-MRSA) who have not had previous contact with health facilities [5]. More recently, livestock-associated MRSA (LA-MRSA) has also been widely reported among several species of animals including pigs, poultry and cows [6,7,8]. Moreover, it seems that most mammals can be colonized and/or infected by MRSA since it has also been isolated from pets, such as dogs, cats and horses, and several species of free-living animals [9,10]. The ability of *S. aureus* to colonize various host species makes it an increasingly recognized zoonotic pathogen. While some clonal complexes (CCs) of *S. aureus* seem restricted to a certain host, such as ST5 in poultry, other CCs such as CC8, CC22 and CC398 have an extended host spectrum [11]. For instance, initially, *S. aureus* CC130 was only detected in cattle. More recently, it has been repeatedly found in wild animals and humans and is typically associated with the *mec*C gene which confers resistance to methicillin [12]. MRSA strains are resistant to almost all beta-lactam antibiotics due to an alteration in the penicillin-binding protein (PBP2a) that is encoded by the *mec* genes [13]. The *mec* genes are located in the staphylococcal cassette chromosome *mec* (SCCmec) which is characterized as a large and potentially transmissible genetic element that not only carries the *mec* genes but also other antimicrobial resistance genes [14]. SCC*mec* elements are highly diverse and are currently classified into 14 types [15]. Of all the *mec* genes, *mec*A is the predominant variant. However, in 2011, a divergent *mec*A homologue, *mec*C, was identified in MRSA strains from human samples in Ireland [16]. Later, two new *mec* genes were reported, *mec*B and *mec*D, which are much less frequent and were both detected in *Macrococcus caseolyticus* [17,18]. After the first detection of *mec*C*,* it has been reported in several countries of all continents and from multiple origins, including humans, animals and the environment [10,19,20,21,22,23,24,25,26]. The origins and reservoirs of the *mecC* gene in MRSA strains are still unknown. First, *mec*C was associated with LA-MRSA. However, the continued detection of this gene in wild animals and in the environment indicates that the primary reservoir of the *mec*C gene may be the natural environment [10]. The *mec*C gene was first described encoding resistance to methicillin in *S. aureus* over a decade ago. However, although numerous MRSA studies have been published in recent years in Portugal, *mec*C has never been detected. Globalized maritime trading routes facilitate the dispersal of synanthropic rodents and their pathogens, with seaports constituting pivotal entry points and potential hotspots of disease. Depending on the extension of the urban matrix, commensal rodents may constitute important vehicles of MRSA not only to humans (directly or indirectly) but also to other resident species with which they may interact. In this study, we isolated methicillin-susceptible *S. aureus* (MSSA) and MRSA from wild synanthropic rodents captured in two port cities, one continental/highly urbanized and one insular/less urbanized, characterizing all isolates regarding the antimicrobial resistance, virulence and clonal lineages.

## 2. Materials and Methods

### 2.1. Samples and Bacterial Isolates

From May 2019 to March 2020, mouth and rectal swabs samples were recovered from 204 wild rodents, including nine *Mus musculus*, 75 *Rattus rattus* and 120 *Rattus norvegicus*. Rodents were live trapped with Sherman and Tomahawk traps in port and surrounding areas (up to 10 km) of Lisbon and Ponta Delgada (São Miguel island, Azores), Portugal. Animals were obtained in the framework of the R&D project PTDC/SAU-PUB/29254/2017 and all procedures followed the European directive 2010/63/EU as stated by the Animal Welfare Body ORBEA of the Faculty of Sciences, University of Lisbon (ethics committee statement 4/2018). The location and specific characteristics of the animals are shown in the supplementary material (Table S1). One sample was collected from each animal. The samples were incubated in BHI broth (Oxoid, Basingstoke, Hampshire, England) with 6.5% NaCl for 24 h at 37 °C. The inoculum was seeded onto Baird-Parker agar (Oxoid, Basingstoke, Hampshire, England) supplemted with Egg Yolk Tellurite Emulsion, Mannitol Salt agar (Oxoid, Basingstoke, Hampshire, England) and ORSAB (supplemented with 2 mg/L of oxacillin) Oxoid, Basingstoke, Hampshire, England) plates for *S. aureus* and MRSA isolation and incubated at 37 °C for 24–48 h. One colony was recovered from each plate. *S. aureus* species was identified by biochemical tests (Gram staining, DNase and catalase) and confirmed by MALDI-TOF MS (Bruker Daltonics GmbH; Bremen, Germany).

### 2.2. Antimicrobial Susceptibility Testing

The phenotypic resistance characterization of the isolates was performed by the Kirby–Bauer disk diffusion method against the following 14 antimicrobial agents: cefoxitin (30 μg), chloramphenicol (30 μg), ciprofloxacin (5 μg), clindamycin (2 μg), erythromycin (15 μg), fusidic acid (10 μg), gentamicin (10 μg), kanamycin (30 μg), linezolid (10 μg), mupirocin (200 μg), penicillin (1 U), tetracycline (30 μg), tobramycin (10 μg), and trimethoprim/sulfamethoxazole (1.25/23.75 μg). The results were evaluated according to the EUCAST 2018 guidelines with the exception of kanamycin which followed the guidelines of CLSI 2017. *S. aureus* strain ATCC 25923 was used as quality control in the susceptibility assays.

### 2.3. Antimicrobial Resistance and Virulence Genes

All isolates were screened for the presence antimicrobial resistance genes according to their phenotypic resistance. The presence of *mec*A and *mec*C genes was investigated by PCR and sequencing as previously described [27,28]. The following genes were tested: *bla*Z, *bla*Z*-*SCC*mec*XI, *tet*(K), *tet*(M), *tet*(L), *tet*(O), *erm*(A), *erm*(B), *erm*(C), *erm*(T), *msr*(A/B), *mph*C, *lin*A, *lin*B, *vga*A, *vga*B, *vga*C, *aac*(6′)-Ie-*aph*(2″)-Ia*, aph*(3′)-IIIa and *ant*(4′)-Ia [12,29]. The presence of virulence genes encoding Panton–Valentine leucocidin (PVL) (*lukF*/*lukS*-PV), alpha- and beta-hemolysins (*hla* and *hlb*), exfoliative toxins (*eta* and *etb*) and toxic shock syndrome toxin (*tst*) was determined by PCR as previously described [30]. The immune evasion cluster (IEC) system was studied by PCR [31]. The isolates were screened for the presence of the *scn* gene, which is a marker of the IEC system, and the presence of *chp*, *sak*, *sea* and *sep* genes was carried out in *scn*-positive isolates to determine the IEC group. The presence of the genes encoding for SCC*mec* were investigated by PCR as previously described [27,28]. Positive and negative controls used in all experiments belonged to the strain collection of University of Trás-os-Montes and Alto Douro.

### 2.4. Molecular Typing

Multilocus-sequence-typing (MLST) was performed in all isolates and according to Enright et al., 2000 [32]. The sequence type (ST) was obtained by comparing the allelic profile of each isolate to the MLST database. All isolates were typed by *spa*-typing as previously described [33] and the obtained sequences were analyzed using Ridom^®^ Staph-type software (version 1.5, Ridom GmbH, Würzburg, Germany). All isolates were characterized by *agr*-typing (I–IV) using specific primers [34].

## 3. Results

**Figure 1:**
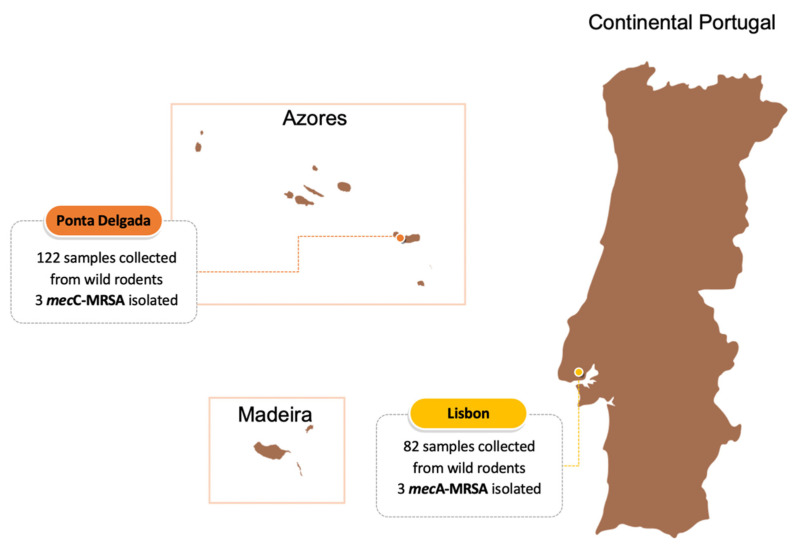
Number of samples collected from wild rodents captured in port areas of Lisbon and Ponta Delgada (S. Miguel island, Azores) and distribution of *mec*A- and *mec*C-MRSA in each location.

From the 204 rodent samples, 38 (18.6%) *S. aureus* were isolated from both *R. norvegicus* and *R. rattus*, 26 in Lisbon and 12 in Ponta Delgada. From the 38 isolates, six (15.7%) MRSA were identified, three in each study city (Figure 1). Three MRSA were *mec*A-positive and three were *mec*C-positive, with the latter only being detected in Ponta Delgada. The characteristics of the isolates are shown in Table 1. *S. aureus* was isolated from 13 (17.3%) and 25 (20,8%) of the 75 and 120 *R. rattus* and *R. norvegicus*, respectively. All *mec*A-MRSA strains were isolated from *R. rattus* whereas two *mec*C-MRSA were isolated from *R. rattus* and one from *R. norvegicus*. All *mec*C-carrying strains were isolated from rodents from S. Miguel island (Azores) and *mec*A-MRSA isolates were recovered from two different Lisbon locations which are located less than 500 m from hospitals (Table S1). Isolates of *mec*C-MRSA showed resistance to penicillin and cefoxitin and harbored the *bla*Z-SCC*mec*XI. They were all ascribed to *spa*-types t1535 and ST1945 (which belongs to CC130), to SCC*mec* type XI and *agr* type III. The *mec*C-positive isolates lacked the virulence genes tested for but were positive for *scn* and *sak* genes from the IEC system and were, therefore, classified as IEC-type E. Two *mec*A-positive isolates showed a multidrug-resistant profile, since they were resistant to at least three different classes of antibiotics. All *mec*A-MRSA were resistant to penicillin, cefoxitin and ciprofloxacin and harbored the *bla*Z gene. Two strains also had resistance to erythromycin and one strain was resistant to aminoglycosides and harbored *aph*(3′)-IIIa; however, it lacked the *aac*(6′)-Ie-*aph*(2″)-Ia gene which confers resistance to gentamicin. Two *mec*A-MRSA isolates were ascribed to ST22 and *spa*-type t747 and one isolate was ST36 (CC30) and t018. Three strains were not typeable with respect to the SCC*mec* types tested and were *agr* type I. These isolates also harbored the *hlb* and *hld* virulence genes. Two MRSA isolates lacked the IEC system genes and one harbored the *scn*, *sak*, *chp* and *sea* and was classified as IEC-type A. Regarding the MSSA isolates, 26 were susceptible to all antibiotics tested. Among the remaining six MSSA, all were resistant to penicillin and harbored the *bla*Z gene. All MSSA isolates, except one, carried the virulence gene *hld* and 22 harbored the *hlb* gene. All strains were negative for *tst*, *eta* and *etb* genes and lacked the PVL toxin. Four MSSA isolates carried the *scn* gene and the IEC genes and were further studied. Two MSSA strains were ascribed to IEC-type C, one to type E, one to type A and one strain harbored only the *scn* gene. Regarding the molecular typing, *agr* I was detected in 18 isolates, nine isolates were *agr* III and five were not typeable. The MSSA isolates were distributed in 11 STs, one new ST first described in this study (ST6574) and another 10 STs, including, ST1094 (*n* = 7), ST130 (*n* = 7), ST398 (*n* = 4), ST5926 (*n* = 3), ST8 (*n* = 3), ST1245 (*n* = 2), ST1318, ST1290, ST34 and ST6. Regarding the *spa*-typing, the MSSA isolates were ascribed to 12 different *spa*-types, including t516 (*n* = 7), t843 (*n* = 7), t1451 (*n* = 6), t4608 (*n* = 2), t3256 (*n* = 2), t1535 (*n* = 2), t2078, t571, t414, t16615, t131 and t19688 which is first reported in this study.

## 4. Discussion

*S. aureus* colonization and infection in wild animals have only been superficially investigated since most research studies focus on other animal species with economic importance [35]. Nevertheless, to fully understand the process of infection and colonization in humans, an ecological approach is required. In fact, the natural colonization of rodents could have a special interest since laboratory rats and mice are commonly used as an experimental model to study *S. aureus* infection [36]. However, studies on the prevalence of *S. aureus* in wild rodents are scarce. In our study, we collected 204 samples of wild rodents living in port and surrounding areas, up to a 10 km radius from the port, both in Lisbon and Ponta Delgada (Azores). These port cities represent different levels of urbanization, with Lisbon portraying a very tight urban matrix in opposition to Ponta Delgada, where, within 2–3 km from the port, a more rural landscape where livestock and farming practices take place. In total, a prevalence of 18.6% of *S. aureus* colonizing these animals was obtained. Six (2.9%) out of 204 samples (detected both in *R. norvegicus* and *R. rattus*) were positive for MRSA, three in Lisbon and three in Ponta Delgada. One study by van de Giessen et al. (2009) reported a prevalence of MSSA and MRSA among rats living on livestock farms of 41.8% and 11.6%, respectively, which was higher than the prevalence of these strains in our study [37]. Raafat et al. (2020) also reported a higher prevalence of *S. aureus* (25.5%) among free-living wild rats but a lower occurrence of MRSA of 1.38% [11]. Nevertheless, other studies have reported a prevalence of MSSA in wild rodents similar to ours [36,38,39]. Three of the six MRSA harbored the *mec*C gene and were typed as ST1945-t1535-*agr*III-SCC*mec*XI. ST1945 belongs to CC130 which carries *mec*C instead of *mec*A. It has been suggested that there might be a mutual exchange of *mec*C-MRSA between livestock and wild animals since it was thought that CC130 originated in ruminants [40]. In fact, one of the three MRSA carrying the *mec*C gene was detected in a *R. norvegicus* captured in a cattle/dairy farm in the outskirts of Ponta Delgada. ST1945-MRSA-t1535 isolates have only been reported in wild animals (red deer, wild rodents and wild birds) in Spain [38,41,42]. Nevertheless, MRSA *spa*-type t1535 has been isolated from humans in several European countries, including Germany and Austria [43,44]. ST1945-MRSA has also been isolated from humans in the UK, Spain and France and from animals in Spain, France and Germany but associated with other *spa*-types [12,45,46,47,48]. ST1945 is usually associated with *spa*-types t1535 or t843 and always related with *mec*C-carrying strains [38,41,42]. Studies have shown that *mec*C-positive ST1945 isolates belong to *agr* III and usually carry the *bla*Z-SCC*mec*XI gene [38,41,42]. All our *mec*C isolates harbored both the *scn* and *sak* genes and they were, consequently, ascribed to IEC type E. Although the presence of IEC genes usually suggests a possible human origin, it has been proposed that IEC-type E might be a conserved trait of ST1945 isolates since several studies conducted in Spain and the UK reported the presence of these genes in *mec*C-MRSA ST1945 isolates [49]. Furthermore, in most studies reporting *mec*C-MRSA, the presence of IEC genes was not investigated. Our isolates were susceptible to all antimicrobial agents tested except for ß-lactams and did not present any of the virulence genes tested as reported in other studies [38,41,42]. The *bla*Z-SCC*mec*XI is an allotype of the *S. aureus bla*Z and has 67% amino acid identity [16]. The origin of the mecC gene is unclear; however, *mec*C-CC130 has been regarded as an animal-adapted lineage of *S*. *aureus* which suggests that *mec*C may have arisen in animals [46]. Therefore, *mec*C-MRSA strains may impose a zoonotic risk with important public health consequences. Although the presence of MRSA in wild animals is not very common, it seems that MRSA isolated from wildlife are more frequently associated with the *mec*C gene since it has been isolated from several animal species, including, foxes, deer, hares, hedgehogs, rodents, otters, rabbits, storks, magpies and vultures [10]. However, we also isolated three *mec*A-positive MRSA in this study. Two *mec*A isolates were ST22-t747-*agr*I and one was ST36-t018-*agr*I. ST22-MRSA-t747 has been reported worldwide, often associated with HA-MRSA. In Portugal, this clone is one of the most frequently found in the nosocomial environment and has been reported associated with several infections [50,51]. The ST22-MRSA-t747 isolate was IEC-type A which may confirm a possible human origin. ST36 belongs to CC30 and it is also a healthcare-associated MRSA clone [52]. MRSA ST36, when associated with SCC*mec* type II, is known as the epidemic clone EMRSA-16 [52]. However, in our study MRSA ST36 was not typeable regarding the SCC*mec*. Several studies have reported HA-MRSA strains colonizing wild animals, particularly those in close proximity with human activities [53,54,55]. In our study, *mec*A-positive strains were isolated in two locations near hospitals which may explain the fact that HA-MRSA strains were identified in wild rodents.

Most of the MSSA isolates from wild rodents had a very low frequency of antimicrobial resistance. In fact, the great majority of the isolates were susceptible to all tested antibiotics. These results are in accordance with other studies conducted in wild animals [41,56]. This low prevalence of antimicrobial resistance determinants in wildlife may be explained by the fact that these animals do not have direct contact with antibiotics and live in the absence of selective pressure [38,56,57]. Nevertheless, other studies have shown that wild animals with no apparent contact with antibiotics carried antimicrobial resistant strains [58,59]. Therefore, wildlife may be considered a sentinel of antimicrobial resistance, environmental pollution and, in consequence, the prevalence of resistance in wild reservoirs will depend on the geographical area where they are living. The most frequently detected resistance in MSSA was to penicillin which was found in six out of 32 isolates. The molecular typing revealed a high diversity of genetic lineages among the MSSA isolates. Eleven STs and 12 *spa*-types were detected. ST1094 and ST130 were the predominant STs found in our study. ST1094 is a singleton and was only found in strains ascribed to *spa*-type t516 and *agr* type I. All ST1094-MSSA-t516 isolates were susceptible to all antibiotics tested with the exception of one penicillin-resistant isolate. ST1094 have been previously reported in wild rodents in Boston, associated with low resistance rates, but all strains were typed as t933 [60]. ST1094 has also been found in Asia in human samples in Myanmar (associated with t516) and in ready-to-eat food in China [61,62]. Five out of seven MSSA ST130 isolates were typed as t843 and two isolates were typed as t3256. CC130 was firstly associated with MSSA but more recently this CC has been continuedly reported as *mec*C-MRSA. CC130 is known to be a livestock-associated lineage particularly common in small ruminants such as domestic sheep and goats [63]. Nevertheless, MSSA CC130-t843 has been isolated from wild animals, including wild rodents and boars [36,37,64]. As for CC130-t3256, this strain has been isolated from humans, bovine mastitis and wild animals, but always in MRSA strains harboring the *mec*C gene [44,65,66]. Four MSSA isolates were ST398 (CC398) of which three were typed as t1451 and one as t571 which seem to be the *spa*-types commonly associated with MSSA CC398 [67]. None of the isolates belonged to the *spa-*type t011 which is the most frequent *spa-*type in CC398 strains [56]. CC398 strains are broadly disseminated across Europe and the rest of the world. Additionally, both CC398 MRSA and MSSA do not seem to have host specificity as they have been isolated from livestock, particularly pigs, but also from humans and wild animals [54,56,68]. In our study, CC398 isolates did not exhibited the antimicrobial resistance patterns, predominantly resistance to tetracycline, often observed in MRSA CC398 and commonly associated with livestock [68]. The *spa*-type t1451 was detected in ST398 strains and also in ST5926 isolates. Interestingly, one ST5926 isolate was not typeable regarding the *agr* and one was *agr* I. Furthermore, both ST5926 isolates were positive for *scn* and *chp* genes, being ascribed to IEC-type C, which points to a possible human origin. One isolate ascribed to ST1318 and *spa*-type t2078 was also positive for IEC genes, namely, *scn* and *sak* (IEC-type E). ST6574 was described in this study for the first time, and it was found in two isolates which were *spa*-type t1535. t1535 has been reported in wild animals associated with *mec*C-MRSA as in our study. The *spa*-type t19688 was also firstly reported in this study and was associated with CC8. Antimicrobial resistance is an important public health problem in both human and veterinary medicine. Epidemiological and surveillance data on new lineages and antimicrobial resistance of *S. aureus* and MRSA will be useful in devising an effective antimicrobial stewardship program in hospitals and also in choosing treatment strategies [69].

## 5. Conclusions

Wild synanthropic rodents are the first *mec*C-positive detected hosts in Portugal. So far, *mec*C-MRSA was only detected in the Azores, but despite the narrow geographic scope of this study, it was found in both *R. norvegicus* and *R. rattus*, in a livestock farm and a forest area, respectively. Furthermore, wild rodents seem to be a natural host of *S. aureus* strains, including MRSA, detected both in continental in insular settings, which can represent a dangerous vector for those strains with zoonotic potential. Besides, rats are considered a common urban pest species, especially in port areas, and their associated pathogens may spread to other animals or humans. Also, the detection of *mec*A-MRSA in *R. rattus* in the Lisbon port is a clear indication of the potential passive worldwide dispersion of these rodents and their zoonotic pathogens, including MRSA, through maritime transport.

## Figures and Tables

**Table 1:** Characteristics of the MRSA and MSSA strains isolated from wild rodents in Portugal.

Isolate	Host Species	Antimicrobial Resistance		Virulence Factors		Molecular Typing			
		Phenotype	Genotype	IEC System	Other Genes	ST (CC)	*spa*	*agr*	SCC*mec*
VS2808	*Rattus rattus*	PEN, FOX	*mec*C, *bla*Z-SCC*mec*XI	Type E	-	1945 (130)	t1535	III	XI
VS2809	*Rattus rattus*	PEN, FOX	*mec*C, *bla*Z-SCC*mec*XI	Type E	-	1945 (130)	t1535	III	XI
VS2810	*Rattus norvegicus*	PEN, FOX	*mec*C, *bla*Z-SCC*mec*XI	Type E	-	1945 (130)	t1535	III	XI
VS2811	*Rattus rattus*	PEN, FOX, CIP, ERY	*mec*A, *bla*Z	*-*	*hlb, hld*	22 (22)	t747	I	N.T.
VS2812	*Rattus rattus*	PEN, FOX, CIP, CN, KAN, ERY, CD	*mec*A*, blaZ, aph*(3′)-IIIa, *erm*A	Type A	*hlb, hld*	36 (30)	t018	I	N.T.
VS2813	*Rattus rattus*	PEN, FOX, CIP	*mec*A, *bla*Z	-	*hlb, hld*	22 (22)	t747	I	N.T.
VS2814	*Rattus norvegicus*	Susceptible	-	-	*hlb, hld*	1094	t516	I	-
VS2815	*Rattus norvegicus*	Susceptible	-	-	*hlb, hld*	1094	t516	I	-
VS2816	*Rattus norvegicus*	PEN	*bla*Z	-	*hlb, hld*	1094	t516	I	-
VS2817	*Rattus rattus*	Susceptible	-	-	*hlb, hld*	1094	t516	I	-
VS2818	*Rattus norvegicus*	Susceptible	-	-	*hlb, hld*	1094	t516	I	-
VS2819	*Rattus rattus*	Susceptible	-	-	*hlb, hld*	1094	t516	I	-
VS2820	*Rattus norvegicus*	Susceptible	*-*	-	*hlb, hld*	1094	t516	I	-
VS2821	*Rattus norvegicus*	Susceptible	*-*	-	*hlb, hld*	130	t843	III	-
VS2822	*Rattus norvegicus*	Susceptible	*-*	-	*hlb, hld*	130	t843	III	*-*
VS2823	*Rattus norvegicus*	Susceptible	*-*	-	*hlb, hld*	130	t843	III	-
VS2824	*Rattus norvegicus*	Susceptible	*-*	*scn*	*hld*	130	t843	III	-
VS2825	*Rattus norvegicus*	Susceptible	*-*	*-*	*hlb, hld*	130	t843	III	-
VS2826	*Rattus rattus*	Susceptible	*-*	*-*	*hlb, hld*	130	t3256	III	-
VS2827	*Rattus norvegicus*	Susceptible	*-*	*-*	-	130	t3256	N.T.	-
VS2828	*Rattus norvegicus*	Susceptible	*-*	*-*	*hlb, hld*	1245	t843	III	-
VS2829	*Rattus rattus*	Susceptible	*-*	*-*	*hlb, hld*	1245	t843	III	-
VS2830	*Rattus norvegicus*	PEN	*bla*Z	*-*	*hld*	398	t1451	I	-
VS2831	*Rattus norvegicus*	PEN	*bla*Z	*-*	*hld*	398	t1451	I	-
VS2832	*Rattus norvegicus*	PEN	*bla*Z	*-*	*hld*	398	t1451	I	-
VS2833	*Rattus norvegicus*	Susceptible	*-*	*-*	*hld*	398	t571	I	-
VS2834	*Rattus rattus*	Susceptible	-	Type C	*hld*	5926	t1451	N.T.	-
VS2835	*Rattus norvegicus*	Susceptible	-	Type C	*hld*	5926	t1451	I	-
VS2836	*Rattus norvegicus*	PEN	*bla*Z	Type E	*hlb, hld*	1318	t2078	I	-
VS2837	*Rattus norvegicus*	Susceptible	-	-	*hld*	8 (8)	t4608	I	-
VS2838	*Rattus norvegicus*	Susceptible	-	-	*hlb, hld*	8 (8)	t19688	I	-
VS2839	*Rattus norvegicus*	Susceptible	-	-	*hlb, hld*	8 (8)	t4608	I	-
VS2840	*Rattus norvegicus*	Susceptible	-	-	*hlb, hld*	6574	t1535	III	-
VS2841	*Rattus norvegicus*	Susceptible	-	-	*hlb, hld*	6574	t1535	N.T.	-
VS2842	*Rattus norvegicus*	PEN	*bla*Z	-	*hlb, hld*	34 (30)	t414	N.T.	-
VS2843	*Rattus rattus*	Susceptible	-	-	*hld*	6 (5)	t16615	I	-
VS2844	*Rattus rattus*	Susceptible	-	-	*hlb, hld*	5926	t1451	N.T.	-
VS2845	*Rattus norvegicus*	Susceptible	-	-	*hlb, hld*	1290 (1)	t131	I	-

Abbreviations. PEN: penicillin, FOX: cefoxitin, CIP: ciprofloxacin, ERY: erythromycin, CN: gentamicin, KAN: kanamycin, CD: clindamycin, N.T. not typeable.

## Data Availability

The data presented in this study are available in Supplementary Material Table S1.

## Supplementary Materials

The following are available online at https://www.mdpi.com/article/10.3390/ani11061537/s1, Table S1: location and specific characteristics of wild rodents captured in port areas of Lisbon and Azores island.
