# Supplementary material for: Antimicrobial Resistance and Genetic Lineages of Staphylococcus aureus from Wild Rodents: First Report of mecC-Positive Methicillin-Resistant S. aureus (MRSA) in Portugal
Source: Animals (Basel). 2021 May 25;11(6):1537. doi: 10.3390/ani11061537 (PMC8229929; doi:10.3390/ani11061537)
Supplement: Supplementary file 1 [file animals-11-01537-s001.zip › animals-1188877-supplementary.pdf]

Table S1. Location and specific characteristics of wild rats captured in port areas of Lisbon and Ponta Delgada (S. Miguel island, Azores) positive for *S. aureus*.

Isolate	*Species*	City	Latitude	Longitude	Date	Sex	Age
**VS2808**	*Rattus rattus*	Ponta Delgada	37.750180	-25.593951	12/11/19	F	Adult
**VS2809**	*Rattus rattus*	Ponta Delgada	37.750180	-25.593951	13/11/19	M	Adult
**VS2810**	*Rattus norvegicus*	Ponta Delgada	37.80241	-25.66391	28/11/19	M	Adult
**VS2811**	*Rattus rattus*	Lisbon	38.693644	-9.226419	04/07/19	M	Adult
**VS2812**	*Rattus rattus*	Lisbon	38.74608	-9.17191	31/07/19	M	Adult
**VS2813**	*Rattus rattus*	Lisbon	38.693644	-9.226419	25/06/19	M	Adult
**VS2814**	*Rattus norvegicus*	Lisbon	38.70691	-9.23245	16/10/19	F	Adult
**VS2815**	*Rattus norvegicus*	Lisbon	38.70691	-9.23245	16/10/19	M	Juvenile
**VS2816**	*Rattus norvegicus*	Lisbon	38.70691	-9.23245	31/10/19	M	Adult
**VS2817**	*Rattus rattus*	Lisbon	38.70691	-9.23245	31/10/19	M	Juvenile
**VS2818**	*Rattus norvegicus*	Lisbon	38.70691	-9.23245	05/11/19	M	Adult
**VS2819**	*Rattus rattus*	Lisbon	38.70691	-9.23245	29/10/19	F	Juvenile
**VS2820**	*Rattus norvegicus*	Lisbon	38.70691	-9.23245	08/11/19	F	Adult
**VS2821**	*Rattus norvegicus*	Lisbon	38.700149	-9.176651	27/06/19	M	Adult
**VS2822**	*Rattus norvegicus*	Lisbon	38.700149	-9.176651	04/07/19	F	Adult
**VS2823**	*Rattus norvegicus*	Lisbon	38.700149	-9.176651	04/07/19	M	Adult
**VS2824**	*Rattus norvegicus*	Lisbon	38.700149	-9.176651	28/06/19	F	Adult
**VS2825**	*Rattus norvegicus*	Lisbon	38.700149	-9.176651	04/07/19	F	Adult
**VS2826**	*Rattus rattus*	Ponta Delgada	37.74768	-25.69987	13/11/19	F	Adult
**VS2827**	*Rattus norvegicus*	Ponta Delgada	37.77244	-25.66917	14/01/20	F	Adult
**VS2828**	*Rattus norvegicus*	Lisbon	38.78422	-9.09148	13/02/20	M	Adult
**VS2829**	*Rattus rattus*	Lisbon	38.78422	-9.09148	27/02/20	M	Adult
**VS2830**	*Rattus norvegicus*	Ponta Delgada	37.74962	-25.68882	18/11/19	F	Adult

**VS2831**	*Rattus norvegicus*	Ponta Delgada	37.74962	-25.68882	18/11/19	M	Adult
**VS2832**	*Rattus norvegicus*	Lisbon	38.756484	-9.161519	07/06/19	M	Adult
**VS2833**	*Rattus norvegicus*	Ponta Delgada	37.74835	-25.69694	18/11/19	M	Juvenile
**VS2834**	*Rattus rattus*	Lisbon	38.695068	-9.225746	02/07/19	F	Adult
**VS2835**	*Rattus norvegicus*	Lisbon	38.7541	-9.1925	20/02/20	F	Juvenile
**VS2836**	*Rattus norvegicus*	Lisbon	38.70691	-9.23245	29/10/19	M	Adult
**VS2837**	*Rattus norvegicus*	Ponta Delgada	37.74962	-25.68882	14/11/19	M	Adult
**VS2838**	*Rattus norvegicus*	Ponta Delgada	37.75223	-25.69187	29/11/19	F	Adult
**VS2839**	*Rattus norvegicus*	Ponta Delgada	37.74962	-25.68882	14/11/19	F	Adult
**VS2840**	*Rattus norvegicus*	Lisbon	38.74608	-9.17191	09/09/19	M	Adult
**VS2841**	*Rattus norvegicus*	Lisbon	38.74608	-9.17191	07/08/19	F	Adult
**VS2842**	*Rattus norvegicus*	Ponta Delgada	37.7977	-25.70772	17/01/20	M	Adult
**VS2843**	*Rattus rattus*	Lisbon	38.7607	-9.09702	12/02/20	M	Juvenile
**VS2844**	*Rattus rattus*	Lisbon	38.697153	-9.229570	05/07/19	M	Adult
**VS2845**	*Rattus norvegicus*	Lisbon	38.703235	-9.177966	10/07/19	M	Juvenile
